# Serial Recall Order of Category Fluency Words: Exploring Its Neural Underpinnings

**DOI:** 10.3389/fpsyg.2021.777838

**Published:** 2022-01-06

**Authors:** Matteo De Marco, Annalena Venneri

**Affiliations:** Department of Life Sciences, Brunel University London, Uxbridge, United Kingdom

**Keywords:** grey matter, semantic memory, verbal fluency, item-level, frequency, typicality, age of acquisition, default-mode network

## Abstract

**Background:** Although performance on the category fluency test (CFT) is influenced by many cognitive functions (i.e., including language, executive functioning and speed of processing), item-level scoring methods of CFT performance might be a promising way to capture aspects of semantic memory that are less influenced by intervenient abilities. One such approach is based on the calculation of correlation coefficients that quantify the association between item-level features and the serial order with which words are recalled (SRO).

**Methods:** We explored the neural underpinnings of 10 of these correlational indices in a sample of 40 healthy adults who completed a classic 1-min CFT and an MRI protocol inclusive of T1-weighted (analysed with voxel-based morphometry) and resting-state fMRI sequences for the evaluation of the default-mode network (DMN). Two sets of linear models were defined to test the association between neural maps and each correlational index: a first set in which major demographic and clinical descriptors were controlled for and a second set in which, additionally, all other 9 correlational indices were regressed out.

**Results:** In the analysis of the DMN, ‘SRO-frequency’, ‘SRO-dominance’ and ‘SRO-body-object interaction’ correlational indices were all negatively associated with the anterior portion of the right temporoparietal junction. The ‘SRO-frequency’ correlational index was also negatively associated with the right dorsal anterior cingulate and the ‘SRO-dominance’ correlational index with the right lateral prefrontal cortex. From the second set of models, the ‘SRO-typicality’ correlational index was positively associated with the left entorhinal cortex. No association was found in relation to grey matter maps.

**Conclusion:** The ability to retrieve more difficult words during CFT performance as measured by the correlational indices between SRO and item-level descriptors is associated with DMN expression in regions deputed to attentional reorienting and processing of salience of infrequent stimuli and dominance status. Of all item-level features, typicality appears to be that most closely linked with entorhinal functioning and may thus play a relevant role in assessing its value in testing procedures for early detection of subtle cognitive difficulties in people with suspected Alzheimer’s degeneration. Although exploratory, these findings warrant further investigations in larger cohorts.

## Introduction

The Category Fluency Test (CFT) is among the most common neurocognitive instruments. When the classic version of the test (the ‘1-min’ CFT) is administered, participants are asked to generate, over a timed minute, as many words as they can that belong to a target category, such as, among others, *animals*, *vegetables*, *furniture items*, *professions* and *musical instruments* (Reynoso-Alcántara et al., 2019).

The CFT has been widely used as a test of semantic memory in samples with normal and abnormal cognitive abilities (e.g., Nyberg et al., 1996; Sumiyoshi et al., 2018; Venneri et al., 2018). However, a number of studies has described this test and its standard performance score (i.e., the total number of correct entries generated in 1 min) as a measure of linguistic (Whiteside et al., 2016) or executive functioning (Gibbons et al., 2012; Amunts et al., 2021). Experimental evidence indicates that additional functions, such as speed of processing (Elgamal et al., 2011) and episodic memory (Greenberg et al., 2009), may also contribute to CFT scores. This highlights the multidimensional nature of the cognitive skills that sustain performance on this test.

Such multidimensionality, however, leads to an important theoretical consideration that goes beyond the evidence obtained *via* aggregated data: *it is unknown whether the aforementioned cognitive components contribute to CFT performance levels in an equal measure for each individual*. This means that significant variability may exist at a group level in the construct validity of these scores. Clinical researchers interested in the study of semantic memory, however, recognise the potential of the CFT as it is based on free recall (Gruenewald and Lockhead, 1980), is easy and quick to administer in diverse sociocultural contexts (Ardila et al., 2006), and, importantly, is particularly versatile in its scoring and post-processing procedures.

In this respect, a range of methodological approaches has been proposed to obtain novel CFT measures able to capture aspects of score variability more explicitly linked to semantic memory. In particular, the focus on ‘*item-level* semantic features’ is a methodological approach that appears particularly promising. These are descriptors (e.g., word *frequency*, *typicality* and *age of acquisition*) that quantify certain aspects of semantic complexity (e.g., Forbes-McKay et al., 2005; Venneri et al., 2008; Vita et al., 2014; Vonk et al., 2019). One of these approaches has focussed on the correlation between item-level semantic features and serial recall order (SRO), that is the ordinal position within the list of retrieved words (i.e., first word retrieved: SRO = 1; second word retrieved: SRO = 2; third word retrieved: SRO = 3; etc.). Since SRO is a property of memory retrieval, these coefficients of correlation capture the strength of the link between memory retrieval and semantic complexity. Research has shown that, as SRO increases during the 1-min CFT, words that are semantically more difficult tend to be generated (Crowe, 1998; De Marco et al., 2021; Murphy and Castel, 2021). We do not know, however, what neurological mechanisms are at the basis of this phenomenon.

In this observational report, we explored the neural bases of the link between SRO and semantic complexity in a sample of 40 healthy adults. Specifically, we investigated volumetric maps of grey matter and the expression of a major haemodynamic network: the default-mode network (DMN). Of all various large-scale pathways, the DMN was chosen because semantic processing (including retrieval) is one of the types of mental elaboration occurring during rest (Binder et al., 1999), making the DMN an ideal neural candidate to outline the association between neurofunctional resources and SRO-based item-level indices of semantic memory.

## Materials and Methods

### Participants

Forty cognitively-normal adult volunteers (Table 1) were included in this exploratory study (15 males and 25 females). The procedures of recruitment were carried out at the Department of Neuroscience, University of Sheffield (United Kingdom) as an ancillary study to the ‘Virtual Physiological Human: Dementia Research Enabled by IT’ (VPH-DARE@IT) initiative, funded by the EU’s Framework Programme 7. All volunteers were screened by a consultant neurologist and a senior neuropsychologist to rule out the presence of clinical or physiologically-relevant exclusion criteria that would otherwise have an impact on neurocognitive structure or function. Although no specific criterion was set in relation to bi/multilingualism, all participants were monolingual native English speakers who self-described as ‘White British’.

**Table 1:** Demographic and neurocognitive characteristics of the sample.

Descriptive Variable	Mean (SD)	Min	Max	Median	Interquartile Range
Age (years)	66.80 (11.19)	49	85	67	19.75
Education (years)	14.55 (2.71)	10	21	15	4.875
MMSE	27.88 (1.56)	24	30	28	2
CFT (N. Words)	36.58 (6.88)	23	49	37	12.25
CFT (Perseverations)	3.40 (2.76)	0	11	2.5	2
CFT (Intrusions)	0.22 (0.70)	0	4	0	0
Normalised Grey Matter Volume	0.427 (0.052)	0.332	0.526	0.419	0.081

*CFT, Category Fluency Test; MMSE, Mini Mental State Examination; SD, Standard Deviation*.

Each participant completed a session of neurocognitive testing and an experimental brain MRI protocol (acquired using a Philips Ingenia 3 T scanner) consisting of three anatomical and one functional acquisitions. All anatomical sequences (i.e., T1-weighted, T2-weighted and FLAIR) were visually inspected by a neuroradiologist to rule out abnormalities qualifying as exclusion criteria. Additionally, T1-weighted images were also used as part of the experimental procedures (as described in Section “MRI Acquisition and Processing”).

The experimental protocol described in this report received ethical approval from the Health and Care Research Wales Ethics Committee, Ref No: 19/WS/0177. Approval was also obtained from Brunel University London’s Ethics Committee (Ref No 30422-TISS-Jul/2021–33453-2) for retrospective data analysis. Each volunteer signed an informed consent form prior to participation. The study was run in compliance with the 1964 Declaration of Helsinki and subsequent amendments.

### Neurocognitive Testing

Each participant completed a testing session in which the Mini-Mental State Examination and a ‘1-min CFT’ were administered. The former was used in this study as a measure of global cognitive abilities. The latter was administered as part of the experimental procedure. Two categories were used as part of this test: ‘animals’ and ‘fruit’. Two classes of incorrect entries (i.e., ‘perseverations’ and ‘intrusions’) were counted and discarded from any further analyses. As carried out in our previous study on SRO (De Marco et al., 2021), a ‘standardised entry’ was defined for each ‘animal’ and ‘fruit’ to regularise scoring of words having multiple lexical labels (e.g., as with HIPPOPOTAMUS and HIPPO or BUDGERIGAR and BUDGIE) and even out the scores for each item across the sample (see De Marco et al., 2021 for a detailed description of this scoring procedure).

### Item-Level CFT Scoring

Item-level ratings were based on published normative data obtained in cohorts of native English speakers. Each word was scored according to its SRO (i.e., an ordinal variable from 1 to *n*, increasing by one unit for each correct entry generated) and the 10 following item-level semantic properties: typicality (De Marco et al., 2021), age of acquisition (Kuperman et al., 2012), concreteness (Brysbaert et al., 2014), frequency (van Heuven et al., 2014), prevalence (Mandera et al., 2020), recognition time (Mandera et al., 2020), body-object interaction (Pexman et al., 2019), arousal (Warriner et al., 2013), valence (Warriner et al., 2013) and dominance (Warriner et al., 2013). Each of these ten features identifies a distinct aspect of semantic difficulty and, as observed in previous research (De Marco et al., 2021) shows its own unique trend of association with SRO.

**Figure 1:**
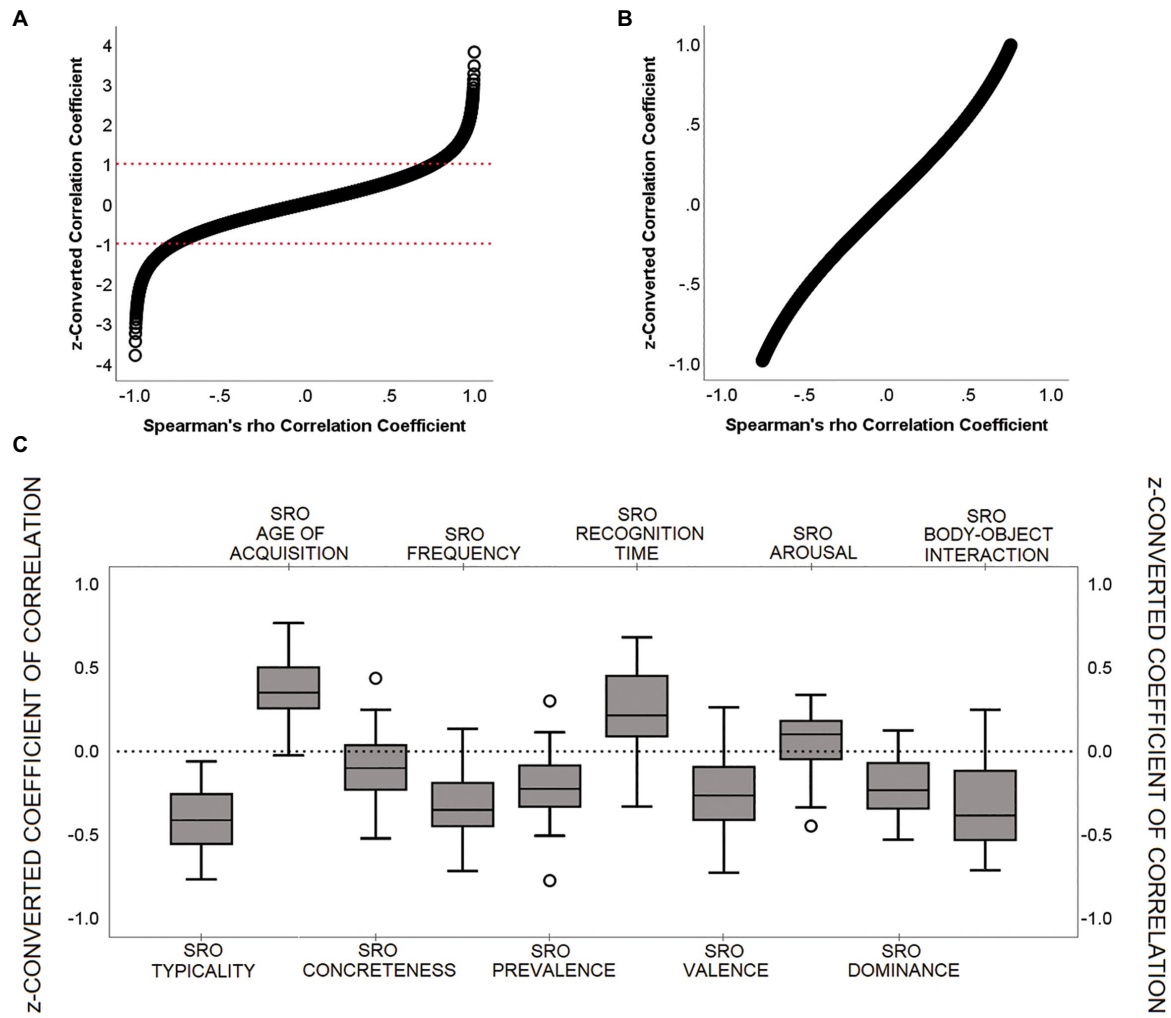
Characterisation of ‘SRO-semantic feature’ *z*-converted correlation coefficients. **(A)** Association between Spearman’s rho (from −0.999 to +0.999) and its *z*-score conversion; **(B)** Detail on the −1 to +1 *z*-score interval; **(C)** Group distribution for each *z*-converted correlation coefficient. All individual scores ranged between −1 and + 1. Within this interval, as can be seen in **(B)**, *z*-conversion does not produce major changes in the score distribution.

Coefficients of correlation (*Spearman’s rho*) were calculated for each participant between SRO and the ten semantic features. In case of missing data due to the absence of normative values for a specific word, this was excluded from the calculation of the correlation coefficient for that specific index. All coefficients were then standardised using a *rho-to-z* conversion (Zar, 2005). To illustrate this conversion more clearly, Figures 1A,B show the association between converted and unconverted coefficients.

### MRI Acquisition and Processing

Three-dimensional T1-weighted images were acquired with the following specifications: 0.94 × 0.94 × 1.00-mm^3^ voxel size, 256 × 256-mm^2^ matrix, 8.2-msec repetition time, 3.84-msec echo delay time, 8° flip angle and a 240 × 240 × 170-mm^3^ field of view.

The functional acquisition was a resting-state echoplanar image devised to measure the brain’s blood oxygenation level-dependent signal for the calculation of the DMN. A 20-sec timeframe of dummy scans was launched prior to each acquisition to enable the scanner to reach electromagnetic equilibrium. The sequence consisted of 125 volumes (each volume was formed by 35 axial contiguous slices) acquired at a 2.6-sec repetition time (total acquisition time: 5 min and 25 sec) and a 35-sec echo delay time, with a 90° flip angle, a 230 × 230 × 140-mm^3^ field of view and a 1.80 × 1.80 × 4.00-mm^3^ voxel size.

Preprocessing and inferential analyses of anatomical images were run with MATLAB (Mathworks Inc., United Kingdom) and Statistical Parametric Mapping 12 (Wellcome Centre for Human Neuroimaging, London, United Kingdom), following the most updated routine of the standard voxel-based morphometry procedures (Ashburner and Friston, 2000). Images were initially segmented to subdivide the native intracranial space into complementary maps of grey matter, white matter and cerebrospinal fluid. Grey matter maps were then normalised and registered to the standard anatomical template available in Statistical Parametric Mapping, modulated and smoothed with an 8-mm full-width at half-maximum Gaussian kernel.

Global native-space tissue-class maps were then quantified in millilitres using the ‘get_totals’ MATLAB command line^1^ to calculate normalised indices of total grey matter (i.e., grey matter volume divided by intracranial volume). This was done to obtain an estimate of retained cortical volume to be used as a proxy of brain reserve. Although quasi-linearly associated with age, in fact, cortical volumes show variability at any given age (Fjell et al., 2014).

Preprocessing and analyses of resting-state fMRI scans were carried out with the CONN toolbox suite (Whitfield-Gabrieli and Nieto-Castanon, 2012). Each image was initially realigned and corrected for in-scanner motion. Spatial displacement was assessed *via* the Artifact Detection Tools on a volume-by-volume basis, to identify outlier dynamics affected by excessive movements (i.e., above the 97th percentile). Each acquisition was then slice-timed, co-registered to its T1-weighted anatomy, normalised to the MNI space and smoothed with a 6-mm full-width at half-maximum Gaussian kernel. A series of denoising steps was then applied. This included band-pass temporal filtering (0.01 Hz – 0.1 Hz), regressing out of the first 10 principal components calculated across the maps of white matter and cerebrospinal fluid (aCompCor; Muschelli et al., 2014) and scrubbing-based censoring of outlier volumes. Additionally, the six linear and rotational rigid-body transformation indices of head motion calculated during realignment, their temporal derivatives and their squared values (for a total of 24 parameters) were also regressed out.

**Figure 2:**
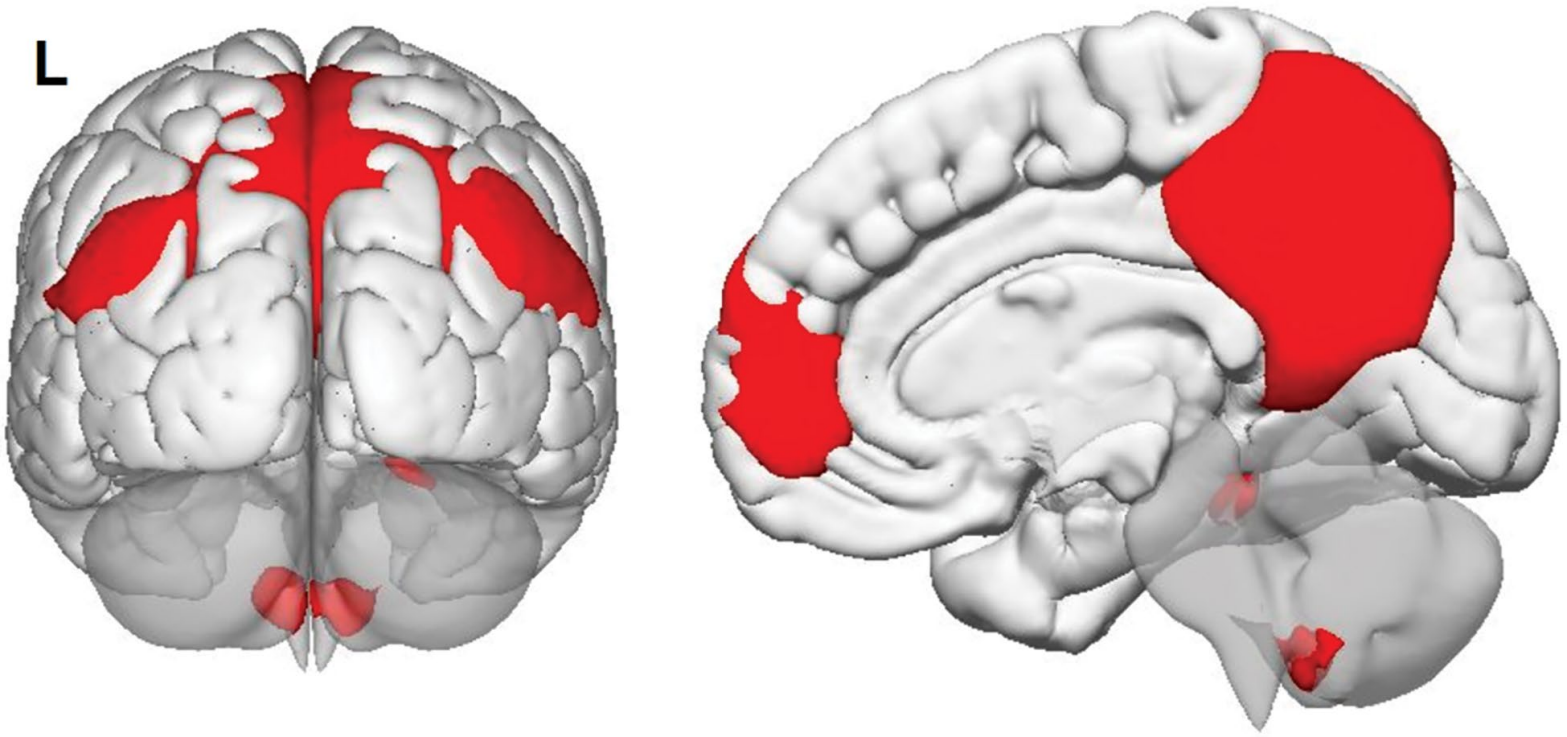
Group-level map of default-mode network in the sample, as calculated by independent component analysis.

A group-level independent component analysis was run to extract individual maps of major large-scale brain networks (Calhoun et al., 2001). The ‘Fast ICA’ optimisation principle and ‘GICA3’ back-reconstruction were applied to calculate 20 independent components. Each group-level map was then inspected to identify that corresponding to the DMN (Figure 2).

### Inferential Models

**Figure 3:**
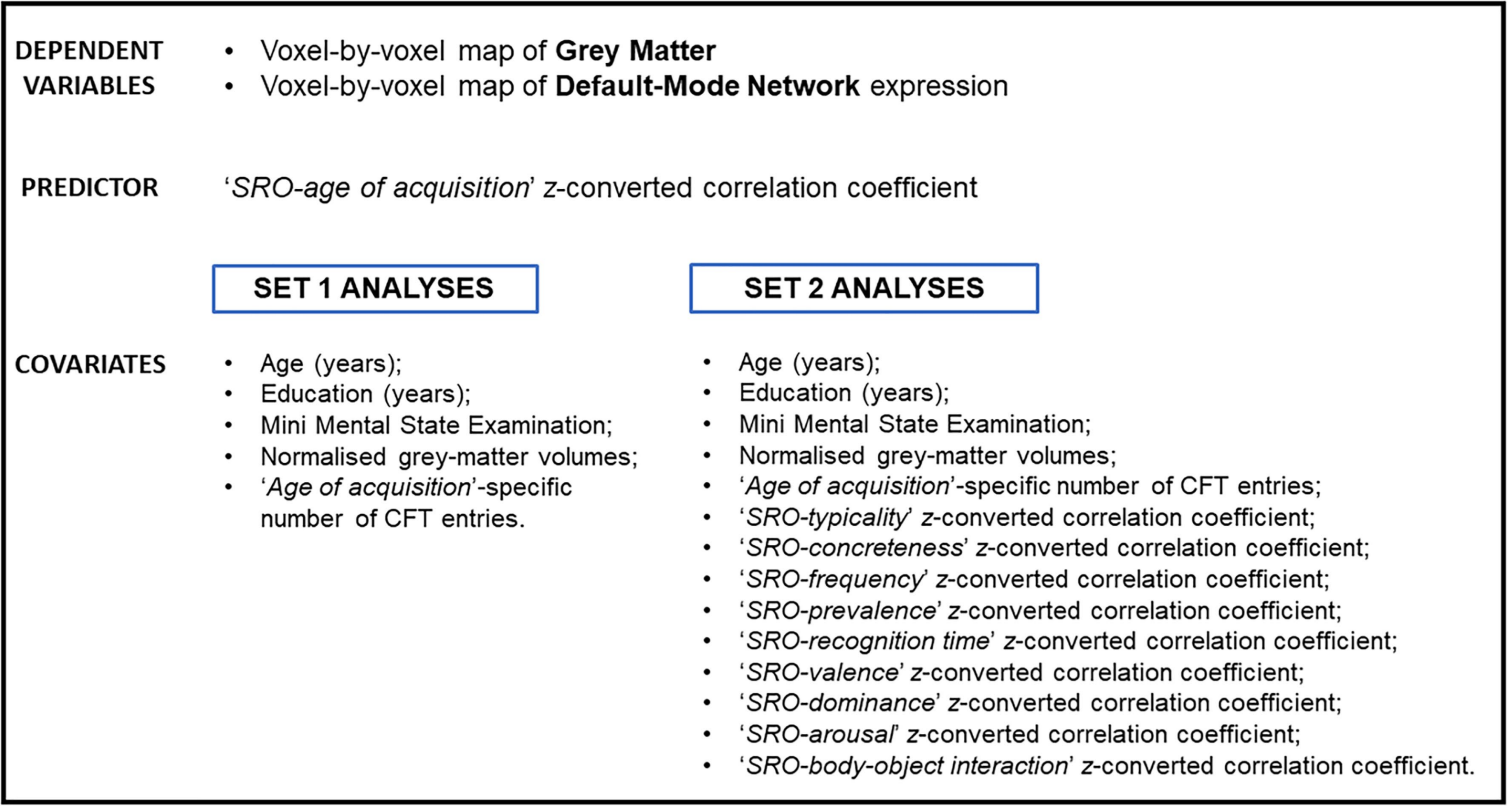
List of variables included in the inferential models of this study. The ‘SRO-age of acquisition’ correlational feature was used as example in this image.

Two sets of analysis were defined. The association between each correlational index and neural maps was tested with a first set of inferential models. In these analyses, whole-brain maps of grey matter and DMN (both expressed as *Beta* scores) were modelled voxel by voxel as a function of each ‘SRO-semantic feature’ *z*-converted correlation coefficient. Each model was corrected for age, years of education, Mini-Mental State Examination score, normalised volumes of total grey matter and number of CFT entries on which that specific feature was calculated (Figure 3). These analyses were thresholded at an uncorrected cluster-forming threshold of *p* < 0.001 as recommended by the literature (Woo et al., 2014). Clusters were deemed significant when surviving a cluster-level Family-Wise Error-corrected *p* < 0.05.

A second set of models was then devised to explore whether any of the correlational indices was particularly informative of the underlying neural architecture above and beyond the predictive effect of all other indices. These linear models were similar to those of the first set of analyses, with the addition of nine further covariates (i.e., the other nine *z*-converted correlational indices; Figure 3). Moreover, a more conservative cluster-forming threshold (an uncorrected *p* < 0.0001) was used in these analyses to capture the strongest effects only.

## Results

All coefficients of correlation were calculated with *n_words_* ≥ 22. The distributions of the ten *z*-converted coefficients of correlation did not breach the assumption of normality (all *Shapiro–Wilk’s* *p*-values > 0.05). Boxplot graphs were created to capture the direction of the ‘SRO-semantic feature’ trend (i.e., positive or negative) and visualise the proportion of participants who showed instead a countertrend (Figure 1C). *z*-converted scores were all between +1 and − 1. The only two coefficients with no apparent dominant direction (i.e., less than 85% of the sample showing a consistent positive/negative trend) were ‘SRO-concreteness’ and ‘SRO-arousal’. Of these, the central tendency of the distribution of the ‘SRO-arousal’ coefficients was not different from zero (*p* = 0.092, one-sample *t*-test).

No associations emerged from the analyses of grey matter volumetric maps.

**Figure 4:**
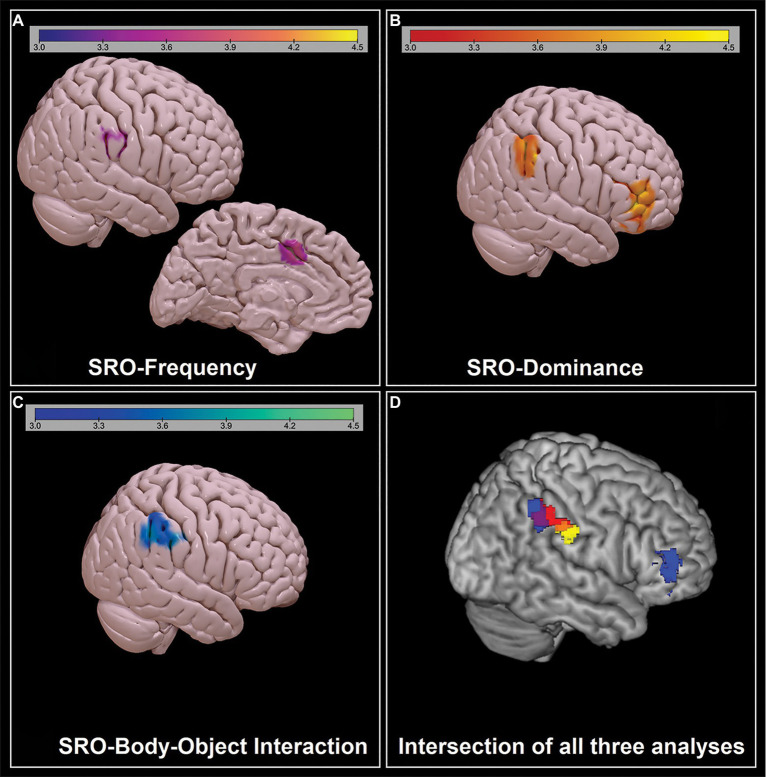
Significant negative associations between SRO-semantic feature *z*-converted correlation coefficients and DMN expression, as emerging from the first set of inferential models: **(A)** ‘SRO-frequency’; **(B)**: ‘SRO-dominance’; **(C)** ‘SRO-body-object interaction’. The strength of the association (expressed in *z*-scores) is reported at the top. **(D)** Intersection of the three maps: For a better visualisation, the clusters are shown at a depth of 8 voxels.

**Figure 5:**
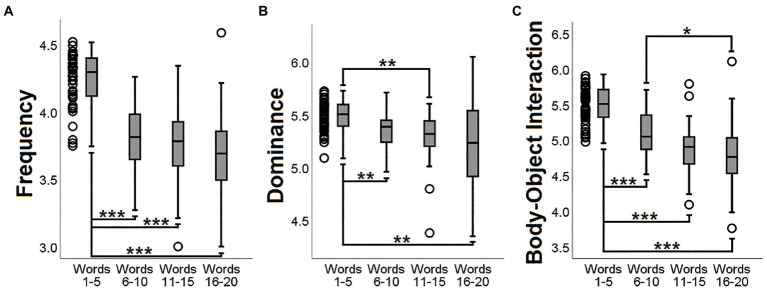
*Post hoc* analyses of downward trends showing significant results. For all three semantic indices (**A**: Frequency; **B**: Dominance; **C**: Body-Object Interaction), the first group of words (illustrated as dots on the left-hand side) was significantly different from the other groups. ^***^p < 0.001; ^**^p < 0.01; ^*^p < 0.05. All *p*-values were Bonferroni-corrected.

Three significant results were found in the first set of analyses of the DMN. The ‘SRO-frequency’ coefficient was negatively associated with the DMN in the right inferior parietal and in the middle-anterior cingulate cortex. The ‘SRO-dominance’ coefficient was negatively associated with the DMN in two clusters located in the right lateral prefrontal and right inferior parietal lobe. Finally, the ‘SRO-body-object interaction’ coefficient was negatively associated with the expression of the DMN in the right inferior parietal lobe. These findings, shown in Figure 4 and described in Table 2, were further explored to characterise the three coefficients in more detail and facilitate interpretation. Performances were split in groups of five words according to their SRO (i.e., ‘Words 1–5’, ‘Words 6–10’, ‘Words 11–15’ and ‘Words 16–20’). All three semantic indices showed a downward trend (as reflected by the negative correlation coefficient), and the first group of five words was significantly different from the others (Figure 5).

**Table 2:** Significant findings emerging from the first set of inferential models.

Cluster-Level *pFWE*	Cluster Extent (voxels)	Peak-Level *z*-Score	Side	BA	Brain Region	Talairach Coordinates		
						x	y	z
**SRO-Dominance – DMN Expression – Negative Association**								
< 0.001	382	4.96	R	40	Inferior Parietal Lobule	53	−39	37
		4.45	R	40	Inferior Parietal Lobule	51	−40	46
		3.97	R	40	Inferior Parietal Lobule	50	−44	46
		3.7	R	40	Supramarginal Gyrus	46	−39	35
0.003	206	4.69	R	46	Inferior Frontal Gyrus	48	39	2
		4.3	R	46	Inferior Frontal Gyrus	42	37	11
		4.27	R	46	Inferior Frontal Gyrus	46	43	9
		4.22	R	10	Inferior Frontal Gyrus	46	43	−2
		3.99	R	46	Middle Frontal Gyrus	38	30	15
		3.86	R	10	Inferior Frontal Gyrus	44	45	1
		3.71	R	11	Middle Frontal Gyrus	42	40	−12
**SRO-Frequency – DMN Expression – Negative Association**								
0.004	195	4.5	R	40	Inferior Parietal Lobule	55	−24	31
		4.11	R	2	Postcentral Gyrus	50	−25	34
0.021	146	4.26	R	32	Cingulate Gyrus	4	25	26
		4.03	R	32	Anterior Cingulate	8	26	24
**SRO-Body-Object Interaction – DMN Expression – Negative Association**								
0.008	176	4.28	R	40	Inferior Parietal Lobule	55	−41	41
		4.09	R	1	Postcentral Gyrus	57	−25	38
		3.66	R	40	Inferior Parietal Lobule	59	−31	40

*FWE, Family-Wise Error; BA, Brodmann area; L, left; R, right*.

Since the pattern of associations was right-lateralised, we tested the possibility that the role of the left hemisphere might have been ‘masked’ by regressing out covariates that embed linguistic abilities. We thus re-ran the three inferential models without controlling for MMSE scores and for the number of valid feature-specific CFT entries. The resulting pattern of findings, however, was substantially unaltered, with no cluster emerging in the left hemisphere.

**Figure 6:**
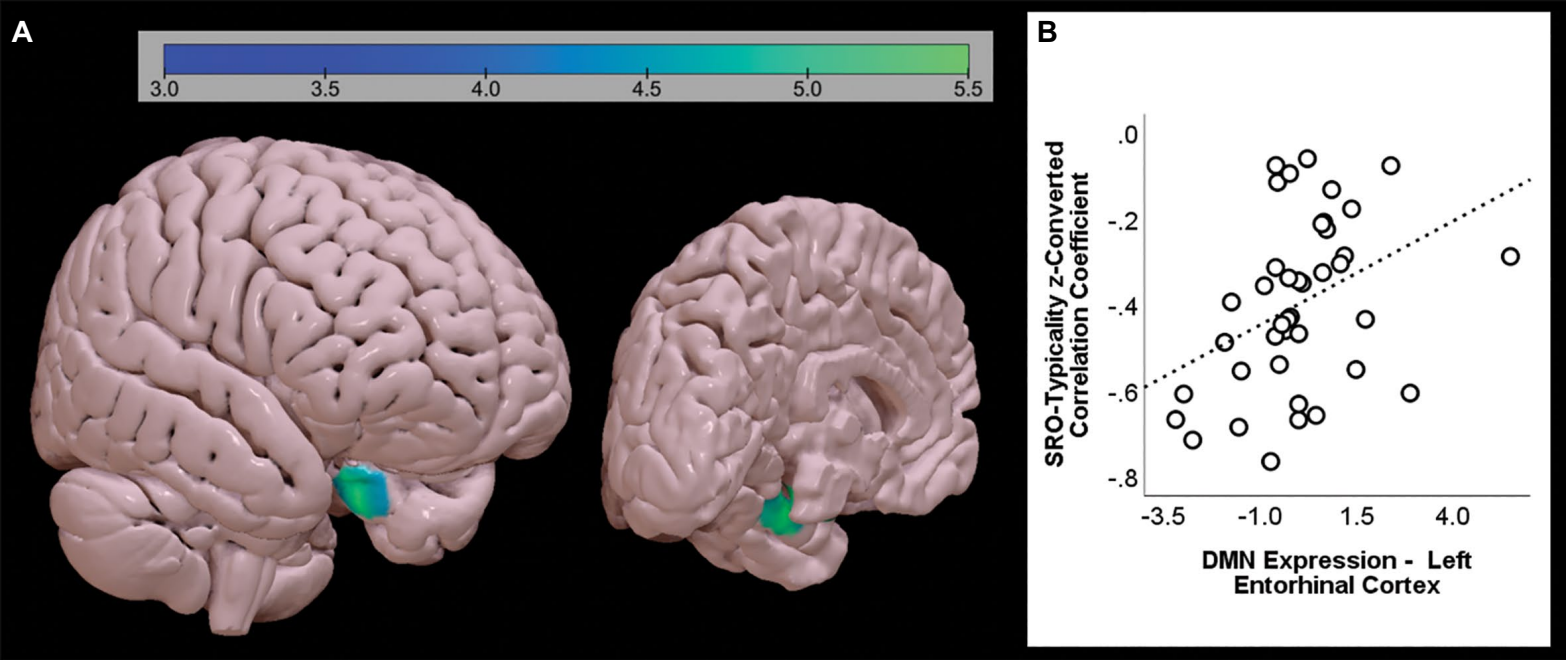
Significant positive association between SRO-typicality and DMN expression, as emerging from the second set of models aimed at identifying the unique pattern of variability accounted for by each index. A single cluster is shown from two different angles **(A)**. The association between the coefficient and the strength of the DMN extracted from the cluster is shown on the right-hand side **(B)**. The graph shows the uncorrected association between the two variables (standardised *β* = 0.386).

One single finding emerged from the second set of models (Figure 6; Table 3). The ‘SRO-typicality’ *z*-converted correlation coefficient was positively associated with DMN expression in the left anterior entorhinal cortex (Brodmann Area 28).

**Table 3:** Significant findings emerged from the second set of inferential models.

Cluster-Level *pFWE*	Cluster Extent (voxels)	Peak-Level *z*-Score	Side	BA	Brain Region	Talairach Coordinates		
						x	y	z
**SRO-Typicality – DMN Expression – Positive Association**								
0.017	48	5.10	L	28	Uncus	−14	−9	−26

*FWE, Family-Wise Error; BA, Brodmann area; L, left*.

## Discussion

This report describes the findings of an exploratory study designed to expand the study of item-level features of CFT words. We modelled maps of grey matter density and DMN expression to infer the statistical association between these neural resources and a series of correlational indices informative of the tendency shown by participants to generate more difficult words as the task progresses. Ten semantic features were studied (investigating ten distinct aspects of semantic difficulty) and four models (three from the first and one from the second set of analyses) yielded significant results.

‘SRO-frequency’, ‘SRO-dominance’ and ‘SRO-body-object interaction’ were the three indices yielding significant findings in the first set of models. As shown in Figure 1C, the distribution of *z*-converted coefficients for these three indices had all a negative sign, meaning that, with increasing SRO, words were characterised by increasingly lower frequency, lower dominance and lower body-object interaction. In all three analyses an association was found in proximity (although slightly more anteriorly) of one of the major DMN hubs, the right inferior parietal lobule (Figure 4D), while the role of the anterior cingulate and right inferior frontal cortex emerged from the analyses of ‘SRO-frequency’ only and ‘SRO-dominance’ only, respectively.

Of the three aforementioned features, frequency has received the most attention from clinical researchers interested in the study of the CFT. Patients with neurodegenerative conditions, such as AD or semantic dementia, in fact, tend to generate words of significantly higher frequency of use than those generated by normal controls (Binetti et al., 1995; Marczinski and Kertesz, 2006). Moreover, when the focus is on SRO, words generated by normal adults tend to be of increasingly lower frequency as the task progresses (Crowe, 1998; De Marco et al., 2021; Murphy and Castel, 2021) and this has also been described in patients with AD (Vonk et al., 2019). In this respect, our results indicate that the steeper this decline, the more strongly expressed the DMN. As shown in Figure 5, frequency of words retrieved at the start of the performance (i.e., Words 1–5) was significantly higher than that of later words. All but two participants had a negative ‘SRO-frequency’ correlation coefficient (see the Supplementary Material for more details), an observation that indicates a more effortful retrieval from Word-6 onwards.

More distinctively than frequency, dominance and body-object interaction are two aspects of semantic processing that embody a significant degree of perceived influence on and interplay with the referent. While body-object interaction can be defined as the sensorimotor information conveyed by the word (Hargreaves et al., 2012), dominance is an affective measure of ‘perceived control/power’ transmitted by the item, that is with ratings ranging from ‘*controlled*’ to ‘*in control*’ (Warriner et al., 2013). Both normative studies (i.e., body-object interaction: Pexman et al., 2019; dominance: Warriner et al., 2013) were based on a 1 to 7 rating scale and, although the degree of interactivity of animals and fruits is likely to be linked to their elicited sense of control, the two features are independent from one another, despite their semantic contiguity; for example, entries exist with disproportionately higher body-object interaction than dominance: SPIDER (i.e., scores of 5.32/7 and 3.74/7, respectively) and RAT (i.e., scores of 4.61/7 and 3.35/7, respectively) and words with disproportionately lower body-object interaction than dominance: CONDOR (i.e., scores of 3.35/7 and 6.06/7, respectively) and BULL (i.e., scores of 3.78/7 and 6.89/7, respectively). As a result, the correlation between the two features calculated for all entries with valid normative scores (i.e., *n* = 198) generated as part of our previous study (De Marco et al., 2021) was only moderate, (i.e., *rho* = 0.418). The degree of interactivity supports ‘*the process of generating meaning*’ (Tousignant and Pexman, 2012). Thus, words with higher body-object interaction scores tend to be easier exemplars of their reference category. In a similar way, words eliciting a stronger sense of control/power tend to stand out more easily within a category. As a result, as with ‘SRO-frequency’, stronger negative correlations for both ‘SRO-body-object interaction’ and ‘SRO-dominance’ indicated an ability to retrieve words of increasingly semantic difficulty during the course of the test.

The role of the anterior portion of the inferior parietal lobule was the common denominator of all three SRO-related trends (Figure 2D). This territory is part of the right temporoparietal junction, a region that has been widely studied in relation to perspective taking in social cognition (Martin et al., 2020; Valera-Bermejo et al., 2021) and episodic memory processing (De Marco et al., 2019). This region, however, serves a second major function, that is attentional reorienting (Corbetta et al., 2008; Mitchell, 2008). The exploration of a semantic category under the constraints imposed by the CFT requires a shift from automatic to controlled semantic retrieval in order to explore the category in more depth and access more difficult exemplars during the course of the 1-min time limit (Crowe, 1996, 1998; Hurks et al., 2004, 2006; Del Hoyo et al., 2015). To achieve this, attentional resources help disengage from semantic clusters that have been exhausted and shift to other unexplored clusters, or towards certain descriptors that might act as a cue (e.g., animals with antlers, animals that live in polar regions, or fruits with edible peel). The right temporoparietal junction, in particular, is involved when there is a conscious use of a cue to reorient attention, but not when this is done without a cue (Wilterson et al., 2021). Although it is not possible to infer causal relationships from these findings, individuals with stronger DMN expression in the right anterior inferoparietal lobule may have an advantage at exploring semantic categories with more flexibility thanks to attentional resources deployed to scan the target category in more detail.

The ‘SRO-frequency’ coefficient was also associated with DMN expression in the dorsal anterior cingulate cortex (Figure 1A). This section of the anterior cingulate is functionally coupled with an ensemble of regions that includes the insula, bilaterally, the anterior prefrontal cortex bilaterally, and the anterior portion of the right inferior parietal lobule (Margulies et al., 2007). This pattern includes the right parietal territory emerged from our analyses as well as the salience network (Menon and Uddin, 2010). The salience network is typically anticorrelated with the DMN, but inter-network connectivity positively supports memory performance in normal individuals (Van Hoorem et al., 2018), suggesting that enhanced DMN expression in the dorsal anterior cingulate might confer a computational advantage. Of all semantic features, such advantage would emerge in relation to frequency because the dorsal anterior cingulate plays a role in detection of salient and *infrequent* stimuli (Manza et al., 2016).

The third and final territory where significant results were found from the first set of analyses was the right frontal lobe, in a cortical cluster extending between the inferior and middle frontal gyri and encompassing a number of peaks within the dorsolateral prefrontal territory and a large portion of the ventrolateral prefrontal cortex (Figure 1B). DMN expression in this region was associated with the standardised coefficient of correlation between SRO and dominance, that is with the tendency to generate progressively less powerful/dominant entries. While the right dorsolateral prefrontal cortex is a region deputed to executive control, the right ventrolateral prefrontal cortex sustains a multitude of diverse cognitive skills, such as controlled memory retrieval (Kostopoulos and Petrides, 2003), spatial navigation (Carrieri et al., 2018) and management of semantic knowledge (Schendan, 2012). In addition to these general abilities, this area is deputed to the elaboration of cues indicative of the stimulus’ dominant/submissive status (Marsh et al., 2009), a specific skill conceptually aligned with the ability to retrieve words characterised by certain levels of dominance/submissiveness. These findings suggest that the strength of DMN expression in the right lateral prefrontal cortex might support semantic memory during CFT and help retrieve animals and fruits that are more difficult exemplars of their category when described in terms of their perceived dominance.

Overall, this pattern of findings suggests that the DMN is linked to SRO and item-level features *via* mechanisms ascribable to semantic control. Attentional reorientation and detection of salience linked to infrequent or dominant/submissive stimuli, in fact, are all aspects linked to management and manipulation of semantic information. In support of this suggestion, in fact, evidence indicates that semantic control is sustained by frontal and temporoparietal regions (Lambon-Ralph et al., 2017).

Although this interpretation provides a literature-informed coherent view of our findings, it is important to understand why the remaining 7 of the 10 correlational measures showed no significant association with DMN expression. One of the features, ‘concreteness’, was not expected to play any relevant role since all animals and fruit are equally concrete. The variance for concreteness ratings was in fact very low (*s^2^* = 0.07) and the least concrete words had all a second meaning that likely influenced the rating (e.g., MANDARIN, SWALLOW, DATE and GRIZZLY). Other two features associated with non-significant findings were ‘prevalence’ and ‘recognition time’. These are dimensions that characterise word difficulty during recognition and, for this reason, they may not play an equally central role when words are freely recalled (i.e., see Mandera et al. (2020) for a description of data collection procedures implemented to collect normative data for these two features). As for ‘arousal’, the ‘SRO-arousal’ coefficient of correlation did not differ from zero, indicating no directional trend. The trends associated with the last three features (i.e., ‘valence’, ‘age of acquisition’ and ‘typicality’) were, overall, well-defined in terms of directionality (Figure 1C), but it is possible that substantial inter-individual variability exists in the neural processes that sustain this type of elaboration. In our previous study, for instance, we found that the ‘SRO-valence’ association is significantly influenced by age and, therefore, age-dependent mechanisms may exist at a neural level in support of the elaborations linked to pleasantness of stimuli (De Marco et al., 2021). Similarly, age is known to have an effect on age of acquisition of words (Hodgson and Ellis, 1998; Sirois et al., 2006), and, for this reason, there might be substantial age-dependent variability in the way retrieval is linked to these item-level features. In this respect, the findings reported in this study are sufficiently robust to survive statistical significance after correction for major demographic variables known to have an effect on neural mechanisms (i.e., age included).

One only significant cluster survived the second, more stringent set of analyses. The ‘SRO-typicality’ *z*-converted coefficient was positively associated with the anterior portion of the left entorhinal cortex after controlling for all the other nine *z*-converted correlational indices. This association, however, was in the opposite direction, that is the increasingly less typical the exemplars, the *weaker* DMN expression in the entorhinal cortex. It is important to point out, however, that all 40 participants showed a negative ‘SRO-typicality’ correlation (i.e., ranging from −0.06 to −0.76) and the positive association found in this analysis does not reflect an increasingly stronger link but, rather, a less steep decline. The entorhinal cortex is a portion of the anterior parahippocampal gyrus that, together with the perirhinal cortex, supports retrieval of ‘context-free’ semantic information (Mishkin et al., 1997). In patients with Alzheimer’s disease, the average typicality of words generated during the CFT is inversely associated with grey matter density in the parahippocampal gyrus, bilaterally, that is the less typical the words on average, the larger the volume in this region (Venneri et al., 2008). The outcome of our analyses suggests that, in healthy adults, the stronger the DMN in an area deputed to semantic retrieval, the more gradual and less steep the tendency to retrieve increasingly atypical exemplars. Despite the size of the sample being limited to 40 adults, this statistical association is particularly robust since it survives a very stringent cluster-forming threshold and it emerges after controlling for all other correlational measures.

Although the interpretational remarks made in this section are informed by the literature and can, in turn, inform the definition of future experimental hypotheses, they are by no means conclusive, i.e., see the debate on the ‘reverse inference fallacy’, whereby cognitive interpretations of functional neuroimaging data should be drawn with caution (Poldrack, 2006; Hutzler, 2014). It is fair to recognise that mechanisms other than those proposed here may have contributed to the above pattern of inferential evidence. The DMN is a neural system that operates ‘as a whole’ and, although the right temporoparietal region does sustain a specific set of functions (as informed by the aforecited publications), it remains possible that its key contribution may occur at a ‘network level’, by supporting network functioning in a more general (rather than specific) way. On a comparable note, more general aspects of CFT performance may have contributed to the individual definition of SRO profiles. For instance, inter-individual differences may exist in how automatic and controlled semantic processes are integrated during test performance and, for this reason, aggregated data might in part mask subject-specific aspects of the ‘automatic-controlled’ interplay. It is for this reason that further studies should be designed with larger cohorts and wider sets of variables, in order to put these findings in a more appropriate context and evaluate the role of alternative interpretations and hypotheses.

In summary, this study expands research in the field of semantic memory by exploring the neural underpinnings of item-level semantic features of words retrieved during the CFT in relation to their SRO. It is paramount to define novel approaches to the study of semantic memory, because this function is informative in clinical settings. Particularly, there is a strong body of evidence emerging from single-case studies (Garrard et al., 2005; van Velzen and Garrard, 2008; Berisha et al., 2015) as well as large cohort longitudinal investigations (Snowdon et al., 1996; Amieva et al., 2008; Gustavson et al., 2020; Payton et al., 2020) indicating that declining semantic memory is among the earliest neurocognitive changes observed along the timeline of Alzheimer’s disease, at a stage when no objective measurable alteration of episodic memory performance is yet detectable. Although this study is only exploratory in nature and needs to be replicated with larger cohorts, it points at the link between aspects of the DMN and semantic control as a potential major drive behind the link between SRO and item-level semantic features.

## Data Availability Statement

The raw data supporting the conclusions of this article will be made available by the authors, without undue reservation.

## Ethics Statement

The studies involving human participants were reviewed and approved by Health and Care Research Wales Ethics Committee (Ref No 19/WS/0177) and by Brunel University London Ethics Committee (Ref No 30422-TISS-Jul/2021-33453-2). The patients/participants provided their written informed consent to participate in this study.

## Author Contributions

MDM contributed to conception and design of the study, acquisition of funding, collected and scored all data, organised the database, performed the statistical analysis and wrote the first draft of the manuscript. AV contributed to acquisition of funding, writing and revision of the manuscript. All authors agreed to be accountable for the content of this work.

## Funding

This research was supported by Neurocare (United Kingdom), under Grant agreement No. 181924 to MDM and AV, and by Alzheimer’s Research UK, under the Pump Priming Grant scheme to MDM. The acquisition of neuroradiological data was supported by funding from the European Union Seventh Framework Programme (FP7/2007e2013) under grant agreement no. 601055, VPH-DARE@IT to AV.

## Conflict of Interest

The authors declare that the research was conducted in the absence of any commercial or financial relationships that could be construed as a potential conflict of interest.

## Publisher’s Note

All claims expressed in this article are solely those of the authors and do not necessarily represent those of their affiliated organizations, or those of the publisher, the editors and the reviewers. Any product that may be evaluated in this article, or claim that may be made by its manufacturer, is not guaranteed or endorsed by the publisher.

## Footnotes

^1^ http://www0.cs.ucl.ac.uk/staff/gridgway/vbm/get_totals.m

## References

[ref1] Amieva H. Le Goff M. Millet X. Orgogozo J. M. Pérès K. Barberger-Gateau P.. (2008). Prodromal Alzheimer’s disease: successive emergence of the clinical symptoms. Ann. Neurol. 64, 492–498. doi: 10.1002/ana.21509, PMID: 19067364

[ref2] Amunts J. Camilleri J. A. Eickhoff S. B. Patil K. R. Heim S. von Polier G. G.. (2021). Comprehensive verbal fluency features predict executive function performance. Sci. Rep. 11:6929. doi: 10.1038/s41598-021-85981-1, PMID: 33767208 PMC7994566

[ref3] Ardila A. Ostrosky-Solís F. Bernal B. (2006). Cognitive testing toward the future: the example of semantic verbal fluency (ANIMALS). Int. J. Psychol. 41, 324–332. doi: 10.1080/00207590500345542

[ref4] Ashburner J. Friston K. J. (2000). Voxel-based morphometry--the methods. Neuroimage 11, 805–821. doi: 10.1006/nimg.2000.0582 10860804

[ref5] Berisha V. Wang S. LaCross A. Liss J. (2015). Tracking discourse complexity preceding Alzheimer’s disease diagnosis: a case study comparing the press conferences of Presidents Ronald Reagan and George Herbert Walker Bush. J. Alzheimers Dis. 45, 959–963. doi: 10.3233/jad-142763, PMID: 25633673 PMC6922000

[ref6] Binder J. R. Frost J. A. Hammeke T. A. Bellgowan P. S. Rao S. M. Cox R. W. (1999). Conceptual processing during the conscious resting state: a functional MRI study. J. Cogn. Neurosci. 11, 80–95. doi: 10.1162/089892999563265, PMID: 9950716

[ref7] Binetti G. Magni E. Cappa S. F. Padovani A. Bianchetti A. Trabucchi M. (1995). Semantic memory in Alzheimer’s disease: an analysis of category fluency. J. Clin. Exp. Neuropsychol. 17, 82–89. doi: 10.1080/13803399508406584 7608305

[ref8] Brysbaert M. Warriner A. B. Kuperman V. (2014). Concreteness ratings for 40 thousand generally known English word lemmas. Behav. Res. Methods 46, 904–911. doi: 10.3758/s13428-013-0403-5, PMID: 24142837

[ref9] Calhoun V. D. Adali T. Pearlson G. D. Pekar J. J. (2001). A method for making group inferences from functional MRI data using independent component analysis. Hum. Brain Mapp. 14, 140–151. doi: 10.1002/hbm.1048, PMID: 11559959 PMC6871952

[ref10] Carrieri M. Lancia S. Bocchi A. Ferrari M. Piccardi L. Quaresima V. (2018). Does ventrolateral prefrontal cortex help in searching for the lost key? Evidence from an fNIRS study. Brain Imaging Behav. 12, 785–797. doi: 10.1007/s11682-017-9734-7, PMID: 28600742

[ref11] Corbetta M. Patel G. Shulman G. L. (2008). The reorienting system of the human brain: from environment to theory of mind. Neuron 58, 306–324. doi: 10.1016/j.neuron.2008.04.017, PMID: 18466742 PMC2441869

[ref12] Crowe S. F. (1996). The performance of schizophrenic and depressed subjects on tests of fluency: support for a compromise in dorsolateral prefrontal functioning. Aust. Psychol. 31, 204–209. doi: 10.1080/00050069608260207

[ref13] Crowe S. F. (1998). Decrease in performance on the verbal fluency test as a function of time: evaluation in a young healthy sample. J. Clin. Exp. Neuropsychol. 20, 391–401. doi: 10.1076/jcen.20.3.391.810, PMID: 9845165

[ref14] De Marco M. Blackburn D. J. Venneri A. (2021). Serial recall order and semantic features of category fluency words to study semantic memory in normal ageing. Front. *Aging Neurosci*. 13:678588. doi: 10.3389/fnagi.2021.678588, PMID: 34413764 PMC8370562

[ref15] De Marco M. Ourselin S. Venneri A. (2019). Age and hippocampal volume predict distinct parts of default mode network activity. Sci. Rep. 9:16075. doi: 10.1038/s41598-019-52488-9, PMID: 31690806 PMC6831650

[ref16] Del Hoyo L. Xicota L. Sánchez-Benavides G. Cuenca-Royo A. de Sola S. Langohr K.. (2015). Semantic verbal fluency pattern, dementia rating scores and adaptive behavior correlate with plasma Aβ42 concentrations in Down syndrome young adults. Front. Behav. Neurosci. 9:301. doi: 10.3389/fnbeh.2015.00301, PMID: 26635555 PMC4649024

[ref17] Elgamal S. A. Roy E. A. Sharratt M. T. (2011). Age and verbal fluency: the mediating effect of speed of processing. Can. Geriatr. J. 14, 66–72. doi: 10.5770/cgj.v14i3.17, PMID: 23251316 PMC3516352

[ref18] Fjell A. M. McEvoy L. Holland D. Dale A. M. Walhovd K. B., for the Alzheimer’s Disease Neuroimaging Initiative (2014). What is normal in normal aging? Effects of aging, amyloid and Alzheimer’s disease on the cerebral cortex and the hippocampus. Prog. Neurobiol. 117, 20–40. doi: 10.1016/j.pneurobio.2014.02.004, PMID: 24548606 PMC4343307

[ref19] Forbes-McKay K. E. Ellis A. W. Shanks M. F. Venneri A. (2005). The age of acquisition of words produced in a semantic fluency task can reliably differentiate normal from pathological age related cognitive decline. Neuropsychologia 43, 1625–1632. doi: 10.1016/j.neuropsychologia.2005.01.008, PMID: 16009244

[ref20] Garrard P. Maloney L. M. Hodges J. R. Patterson K. (2005). The effects of very early Alzheimer’s disease on the characteristics of writing by a renowned author. Brain 128, 250–260. doi: 10.1093/brain/awh341, PMID: 15574466

[ref21] Gibbons L. E. Carle A. C. Mackin R. S. Harvey D. Mukherjee S. Insel P.. (2012). A composite score for executive functioning, validated in Alzheimer’s Disease Neuroimaging Initiative (ADNI) participants with baseline mild cognitive impairment. Brain Imaging Behav. 6, 517–527. doi: 10.1007/s11682-012-9176-1, PMID: 22644789 PMC3684181

[ref22] Greenberg D. L. Keane M. M. Ryan L. Verfaellie M. (2009). Impaired category fluency in medial temporal lobe amnesia: the role of episodic memory. J. Neurosci. 29, 10900–10908. doi: 10.1523/jneurosci.1202-09.2009, PMID: 19726648 PMC2761020

[ref23] Gruenewald P. J. Lockhead G. R. (1980). The free recall of category examples. J. Exp. Psychol. Hum. Learn. Mem. 6, 225–240. doi: 10.1037/0278-7393.6.3.225

[ref24] Gustavson D. E. Elman J. A. Panizzon M. S. Franz C. E. Zuber J. Sanderson-Cimino M.. (2020). Association of baseline semantic fluency and progression to mild cognitive impairment in middle-aged men. Neurology 95, e973–e983. doi: 10.1212/WNL.0000000000010130, PMID: 32606222 PMC7668546

[ref25] Hargreaves I. S. Leonard G. A. Pexman P. M. Pittman D. J. Siakaluk P. D. Goodyear B. G. (2012). The neural correlates of the body-object interaction effect in semantic processing. Front. Hum. Neurosci. 6:22. doi: 10.3389/fnhum.2012.00022, PMID: 22375111 PMC3280593

[ref26] Hodgson C. Ellis A. W. (1998). Last in, first to go: age of acquisition and naming in the elderly. Brain Lang. 64, 146–163. doi: 10.1006/brln.1998.1960, PMID: 9675047

[ref27] Hurks P. P. M. Hendriksen J. G. M. Vles J. S. H. Kalff A. C. Feron F. J. M. Kroes M.. (2004). Verbal fluency over time as a measure of automatic and controlled processing in children with ADHD. Brain Cogn. 55, 535–544. doi: 10.1016/j.bandc.2004.03.003, PMID: 15223200

[ref28] Hurks P. P. M. Vles J. S. H. Hendriksen J. G. M. Kalff A. C. Feron F. J. M. Kroes M.. (2006). Semantic category fluency versus initial letter fluency over 60 seconds as a measure of automatic and controlled processing in healthy school-aged children. J. Clin. Exp. Neuropsychol. 28, 684–695. doi: 10.1080/13803390590954191, PMID: 16723317

[ref29] Hutzler F. (2014). Reverse inference is not a fallacy per se: cognitive processes can be inferred from functional imaging data. Neuroimage 84, 1061–1069. doi: 10.1016/j.neuroimage.2012.12.075 23313571

[ref30] Kostopoulos P. Petrides M. (2003). The mid-ventrolateral prefrontal cortex: insights into its role in memory retrieval. Eur. J. Neurosci. 17, 1489–1497. doi: 10.1046/j.1460-9568.2003.02574.x, PMID: 12713652

[ref31] Kuperman V. Stadthagen-Gonzalez H. Brysbaert M. (2012). Age-of-acquisition ratings for 30,000 English words. Behav. Res. Methods 44, 978–990. doi: 10.3758/s13428-012-0210-4, PMID: 22581493

[ref32] Lambon-Ralph M. A. Jefferies E. Patterson K. Rogers T. T. (2017). The neural and computational bases of semantic cognition. Nat. Rev. Neurosci. 18, 42–55. doi: 10.1038/nrn.2016.150, PMID: 27881854

[ref33] Mandera P. Keuleers E. Brysbaert M. (2020). Recognition times for 62 thousand English words: data from the English crowdsourcing project. Behav. Res. Methods 52, 741–760. doi: 10.3758/s13428-019-01272-8, PMID: 31368025

[ref34] Manza P. Hu S. Chao H. H. Zhang S. Leung H. C. Li C. S. R. (2016). A dual but asymmetric role of the dorsal anterior cingulate cortex in response inhibition and switching from a non-salient to salient action. Neuroimage 134, 466–474. doi: 10.1016/j.neuroimage.2016.04.055, PMID: 27126003 PMC4912860

[ref35] Marczinski C. A. Kertesz A. (2006). Category and letter fluency in semantic dementia, primary progressive aphasia, and Alzheimer’s disease. Brain Lang. 97, 258–265. doi: 10.1016/j.bandl.2005.11.001, PMID: 16325251

[ref36] Margulies D. S. Kelly A. M. C. Uddin L. Q. Biswal B. B. Castellanos F. X. Milham M. P. (2007). Mapping the functional connectivity of anterior cingulate cortex. Neuroimage 37, 579–588. doi: 10.1016/j.neuroimage.2007.05.019 17604651

[ref37] Marsh A. A. Blair K. S. Jones M. M. Soliman N. Blair R. J. R. (2009). Dominance and submission: the ventrolateral prefrontal cortex and responses to status cues. J. Cogn. Neurosci. 21, 713–724. doi: 10.1162/jocn.2009.21052, PMID: 18578604 PMC2735774

[ref38] Martin A. K. Kessler K. Cooke S. Huang J. Meinzer M. (2020). The right temporoparietal junction is causally associated with embodied perspective-taking. J. Neurosci. 40, 3089–3095. doi: 10.1523/jneurosci.2637-19.2020, PMID: 32132264 PMC7141886

[ref39] Menon V. Uddin L. Q. (2010). Saliency, switching, attention and control: a network model of insula function. Brain Struct. Funct. 214, 655–667. doi: 10.1007/s00429-010-0262-0, PMID: 20512370 PMC2899886

[ref40] Mishkin M. Suzuki W. A. Gadian D. G. Vargha-Khadem F. (1997). Hierarchical organization of cognitive memory. *Philos*. Trans. R. Soc. Lond. B Biol. Sci. 352, 1461–1467. doi: 10.1098/rstb.1997.0132, PMID: 9368934 PMC1692056

[ref41] Mitchell J. P. (2008). Activity in right temporo-parietal junction is not selective for theory-of-mind. Cereb. Cortex 18, 262–271. doi: 10.1093/cercor/bhm051 17551089

[ref42] Murphy D. H. Castel A. D. (2021). Age-related similarities and differences in the components of semantic fluency: analyzing the originality and organization of retrieval from long-term memory. Neuropsychol. Dev. Cogn. B Aging Neuropsychol. Cogn. 28, 748–761. doi: 10.1080/13825585.2020.1817844, PMID: 32900273 PMC7937757

[ref43] Muschelli J. Nebel M. B. Caffo B. S. Barber A. D. Pekar J. J. Mostofsky S. H. (2014). Reduction of motion-related artifacts in resting state fMRI using aCompCor. Neuroimage 96, 22–35. doi: 10.1016/j.neuroimage.2014.03.028, PMID: 24657780 PMC4043948

[ref44] Nyberg L. Bäckman L. Erngrund K. Olofsson U. Nilsson L. G. (1996). Age differences in episodic memory, semantic memory, and priming: relationships to demographic, intellectual, and biological factors. J. Gerontol. B Psychol. Sci. Soc. Sci. 51, P234–P240. doi: 10.1093/geronb/51b.4.p234, PMID: 8673644

[ref45] Payton N. M. Rizzuto D. Fratiglioni L. Kivipelto M. Bäckman L. Laukka E. J. (2020). Combining cognitive markers to identify individuals at increased dementia risk: influence of modifying factors and time to diagnosis. J. Int. Neuropsychol. Soc. 26, 785–797. doi: 10.1017/s1355617720000272, PMID: 32207675

[ref46] Pexman P. M. Muraki E. Sidhu D. M. Siakaluk P. D. Yap M. J. (2019). Quantifying sensorimotor experience: body-object interaction ratings for more than 9,000 English words. Behav. Res. Methods 51, 453–466. doi: 10.3758/s13428-018-1171-z, PMID: 30484218

[ref47] Poldrack R. A. (2006). Can cognitive processes be inferred from neuroimaging data? Trends Cogn. Sci. 10, 59–63. doi: 10.1016/j.tics.2005.12.004, PMID: 16406760

[ref48] Reynoso-Alcántara V. Silva-Pereyra J. Vergara-Lope Tristán S. Díaz Camacho J. E. Guiot Vázquez M. I. del Callejo Canal D. D.. (2019). Verbal fluency in Mexican Spanish-speaking subjects with high educational level: ranking of letters and semantic categories. J. Clin. Exp. Neuropsychol. 41, 1001–1014. doi: 10.1080/13803395.2019.1643454, PMID: 31354101

[ref49] Schendan H. E. (2012). “Semantic Memory,” in Encyclopedia of Human Behavior. 2nd Edn. ed. Ramachandran V. S. (Cambridge, MA: Academic Press), 350–358.

[ref50] Sirois M. Kremin H. Cohen H. (2006). Picture-naming norms for Canadian French: name agreement, familiarity, visual complexity, and age of acquisition. Behav. Res. Methods 38, 300–306. doi: 10.3758/bf03192781, PMID: 16956106

[ref51] Snowdon D. A. Kemper S. J. Mortimer J. A. Greiner L. H. Wekstein D. R. Markesbery W. R. (1996). Linguistic ability in early life and cognitive function and Alzheimer’s disease in late life. Findings from the Nun Study. JAMA 275, 528–532. doi: 10.1001/jama.1996.03530310034029, PMID: 8606473

[ref52] Sumiyoshi C. Fujino H. Sumiyoshi T. Yasuda Y. Yamamori H. Fujimoto M.. (2018). Semantic memory organization in Japanese patients with schizophrenia examined with category fluency. Front. Psychiatry 9:87. doi: 10.3389/fpsyt.2018.00087, PMID: 29618990 PMC5871678

[ref53] Tousignant C. Pexman P. M. (2012). Flexible recruitment of semantic richness: context modulates body-object interaction effects in lexical-semantic processing. Front. Hum. Neurosci. 6:53. doi: 10.3389/fnhum.2012.00053, PMID: 22435058 PMC3304254

[ref001] Valera-Bermejo J. M. B. De Marco M. Cerami C. Dodich A. Venneri A. (2021). Large-scale functional networks, cognition and brain structures supporting social cognition and theory of mind performance in prodromal to mild Alzheimer’s disease. Front. Aging Neurosci. 13:766703. doi: 10.3389/fnagi.2021.766703, PMID: 34867292 PMC8636093

[ref54] van Heuven W. J. B. Mandera P. Keuleers E. Brysbaert M. (2014). SUBTLEX-UK: a new and improved word frequency database for British English. Q. J. Exp. Psychol. 67, 1176–1190. doi: 10.1080/17470218.2013.850521, PMID: 24417251

[ref55] van Hoorem R. W. E. Riphagen J. M. Jacobs H. I. L., for the Alzheimer’s Disease Neuroimaging Initiative (2018). Inter-network connectivity and amyloid-beta linked to cognitive decline in preclinical Alzheimer’s disease: a longitudinal cohort study. Alzheimers Res. Ther. 10:88. doi: 10.1186/s13195-018-0420-9, PMID: 30153858 PMC6114059

[ref56] van Velzen M. Garrard P. (2008). From hindsight to insight – retrospective analysis of language written by a renowned Alzheimer’s patient. Interdiscip. Sci. Rev. 33, 278–286. doi: 10.1179/174327908X392852

[ref57] Venneri A. Jahn-Carta C. De Marco M. Quaranta D. Marra C. (2018). Diagnostic and prognostic role of semantic processing in preclinical Alzheimer’s disease. Biomark. Med. 12, 637–651. doi: 10.2217/bmm-2017-0324, PMID: 29896968

[ref58] Venneri A. McGeown W. J. Hietanen H. M. Guerrini C. Ellis A. W. Shanks M. F. (2008). The anatomical bases of semantic retrieval deficits in early Alzheimer’s disease. Neuropsychologia 46, 497–510. doi: 10.1016/j.neuropsychologia.2007.08.026, PMID: 17936858

[ref59] Vita M. G. Marra C. Spinelli P. Caprara A. Scaricamazza E. Castelli D.. (2014). Typicality of words produced on a semantic fluency task in amnesic mild cognitive impairment: linguistic analysis and risk of conversion to dementia. J. Alzheimers Dis. 42, 1171–1178. doi: 10.3233/jad-140570, PMID: 25024315

[ref60] Vonk J. M. J. Flores R. J. Rosado D. Qian C. Cabo R. Habegger J.. (2019). Semantic network function captured by word frequency in nondemented APOE ε4 carriers. Neuropsychology 33, 256–262. doi: 10.1037/neu0000508, PMID: 30489116 PMC6466625

[ref61] Warriner A. B. Kuperman V. Brysbaert M. (2013). Norms of valence, arousal, and dominance for 13,915 English lemmas. Behav. Res. Methods 45, 1191–1207. doi: 10.3758/s13428-012-0314-x, PMID: 23404613

[ref62] Whiteside D. M. Kealey T. Semla M. Luu H. Rice L. Basso M. R.. (2016). Verbal fluency: language or executive function measure? Appl. Neuropsychol. Adult 23, 29–34. doi: 10.1080/23279095.2015.1004574 26111011

[ref63] Whitfield-Gabrieli S. Nieto-Castanon A. (2012). Conn: a functional connectivity toolbox for correlated and anticorrelated brain networks. Brain Connect. 2, 125–141. doi: 10.1089/brain.2012.0073, PMID: 22642651

[ref64] Wilterson A. I. Nastase S. A. Bio B. J. Guterstam A. Graziano M. S. A. (2021). Attention, awareness, and the right temporoparietal junction. Proc. Natl. Acad. Sci. U. S. A. 118:e2026099118. doi: 10.1073/pnas.2026099118, PMID: 34161276 PMC8237657

[ref65] Woo C. W. Krishnan A. Wager T. D. (2014). Cluster-extent based thresholding in fMRI analyses: pitfalls and recommendations. Neuroimage 91, 412–419. doi: 10.1016/j.neuroimage.2013.12.058, PMID: 24412399 PMC4214144

[ref66] Zar J. H. (2005). “Spearman rank correlation: overview,” in Encyclopaedia of Biostatistics. eds. Armitage P. Colton T. (Hoboken, NJ: John Wiley and Sons, Ltd), 1–9.

