# Supplementary material for: Serial Recall Order of Category Fluency Words: Exploring Its Neural Underpinnings
Source: Front Psychol. 2022 Jan 6;12:777838. doi: 10.3389/fpsyg.2021.777838 (PMC8773965; doi:10.3389/fpsyg.2021.777838)
Supplement: Supplementary file 1 [file Table_1.DOCX]

Serial Recall Order of Category Fluency Words: Exploring its Neural Underpinnings

**Matteo De Marco, Annalena Venneri**

Department of Life Sciences, Brunel University London, Uxbridge, UK

***Supplementary Material***

**Supplementary Figure 1**

Four graphs illustrating the trend of the association between Serial Recall Order and word frequency in four participants. (A) and (B) show the two performances with the largest coefficients of correlation, *rho* = -0.713 and *rho* = -0.685, respectively. (C) and (D) show instead the performances of the only two participants with a positive coefficient of correlation, *rho* = 0.029 and *rho* = 0.136, respectively. As can be noted from (D), this participant addressed the task by relying on an unusual letter-by-letter strategy, recalling animals and fruits starting with the letter *A*, then moving on to *B*, then to *C*, etc. Arguably, the use of this strategy undermines the automatic nature of the free recall mode expected from this test and is instead aligned with a more controlled letter-based cued recall. This participant, however, was not excluded from the analyses.

**Supplementary Figure 1**

**
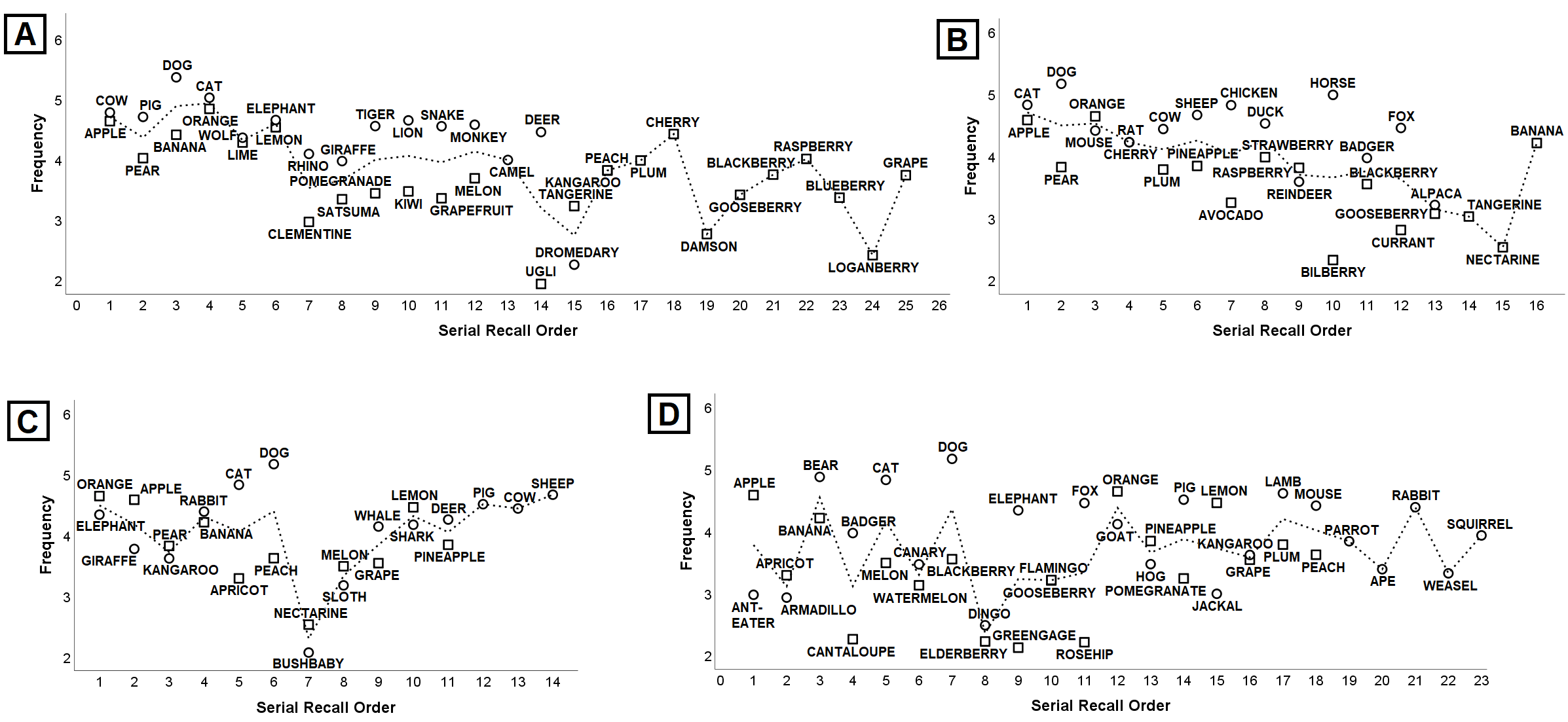
**
